# Supplementary material for: Mechano-regulated metal–organic framework nanofilm for ultrasensitive and anti-jamming strain sensing
Source: Nat Commun. 2018 Sep 19;9:3813. doi: 10.1038/s41467-018-06079-3 (PMC6145903; doi:10.1038/s41467-018-06079-3)
Supplement: Supplementary file 1 — Supplementary Information [file 41467_2018_6079_MOESM1_ESM.pdf]

Mechano Regulated Metal Organic Framework Nanofilm for Ultrasensitive and Anti Jamming Strain Sensing

Pan[1,2,3] et al

[1]CAS Key Laboratory of Magnetic Materials and Devices, Ningbo Institute of Materials Technology and Engineering, Chinese Academy of Sciences, Ningbo, Zhejiang 315201, China

[2]School of Materials Science and Engineering Nanyang Technological University, 50 Nanyang Avenue 639798, Singapore

[3]Zhejiang Province Key Laboratory of Magnetic Materials and Application Technology, Ningbo Institute of Materials Technology and Engineering, Chinese Academy of Sciences, Ningbo 315201, China

All correspondence should be addressed to: liug@nimte.ac.cn (Prof. Gang Liu), chenxd@ntu.edu.sg (Prof. Xiaodong Chen) and runweili@nimte.ac.cn (Prof. Run-Wei. Li)

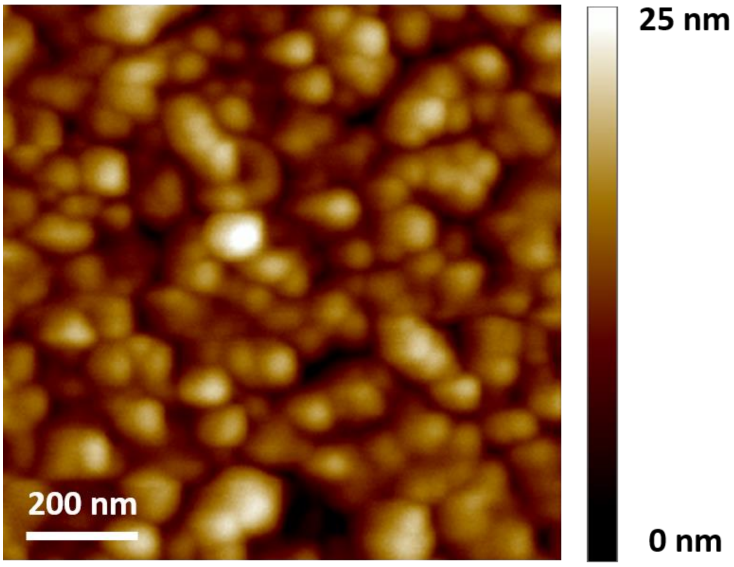

Supplementary Figure 1 │Top-view morphology of the I_2@CuTCA nanofilm. The I_2@CuTCA nanofilm was obtained upon soaking in 100 mM iodine solution and the image was obtained on an atomic force microscope.

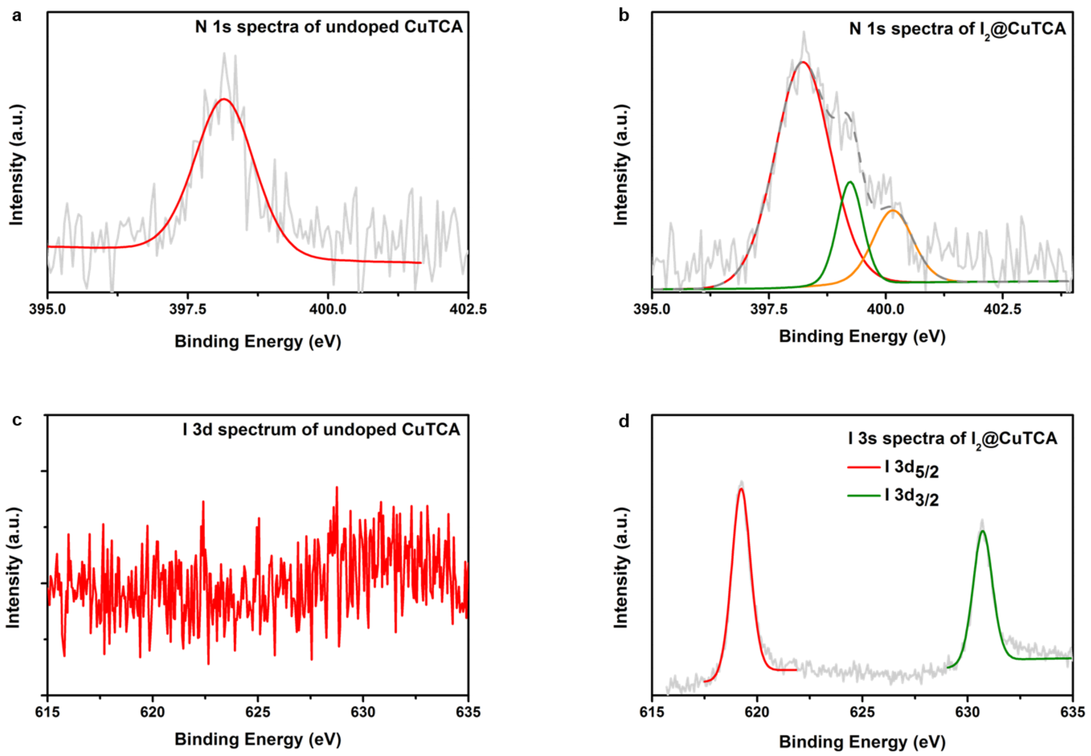

Supplementary Figure 2 │X-ray photoelectron spectra of the MOF films. Corel-level N 1s X-ray photoelectron spectra (XPS) of the (a) pristine CuTCA and (b) iodine doped I_2@CuTCA nanofilms upon soaking in 100 mM iodine solution, respectively. Core-level I 3d XPS spectra of the (c) pristine CuTCA and (d) iodine doped I_2@CuTCA nanofilms upon soaking in 100 mM iodine solution, respectively.

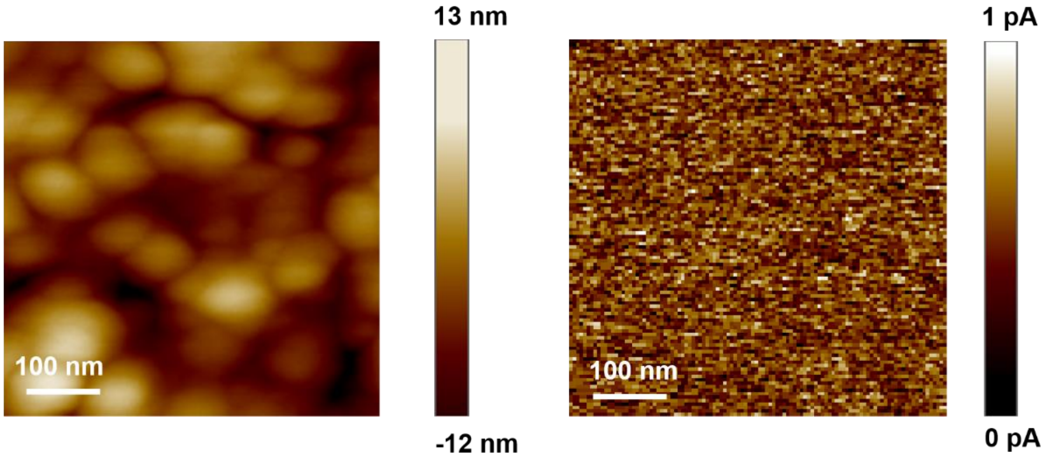

Supplementary Figure 3 | Morphology and current map of the CuTCA nanofilm. The sample is deposited on Au/PET, undoped or unbended.

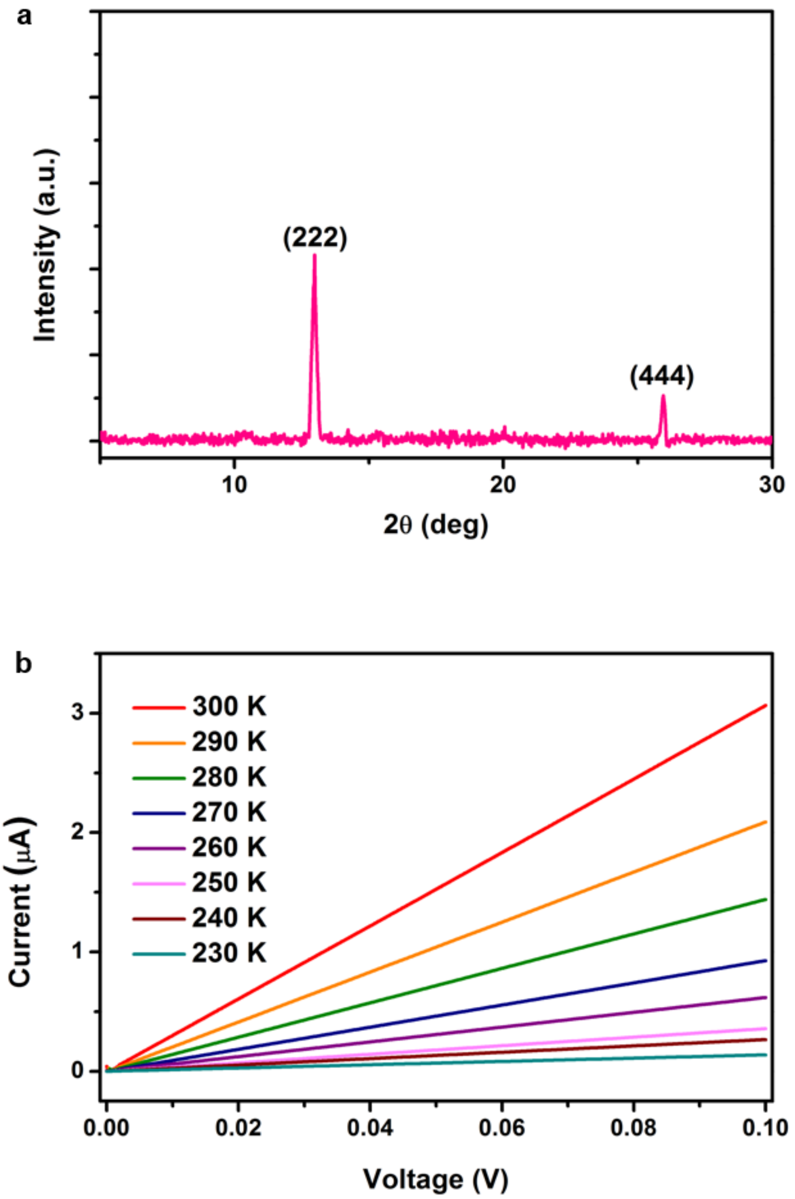

Supplementary Figure 4 | Srtuctural and electrical properties of the MOF film. (a) XRD spectrum and (b) temperature-dependent current-voltage characteristics of the unbended I_2@CuTCA sample with the I_2 molecule/N atom ratio of 1.07:1.

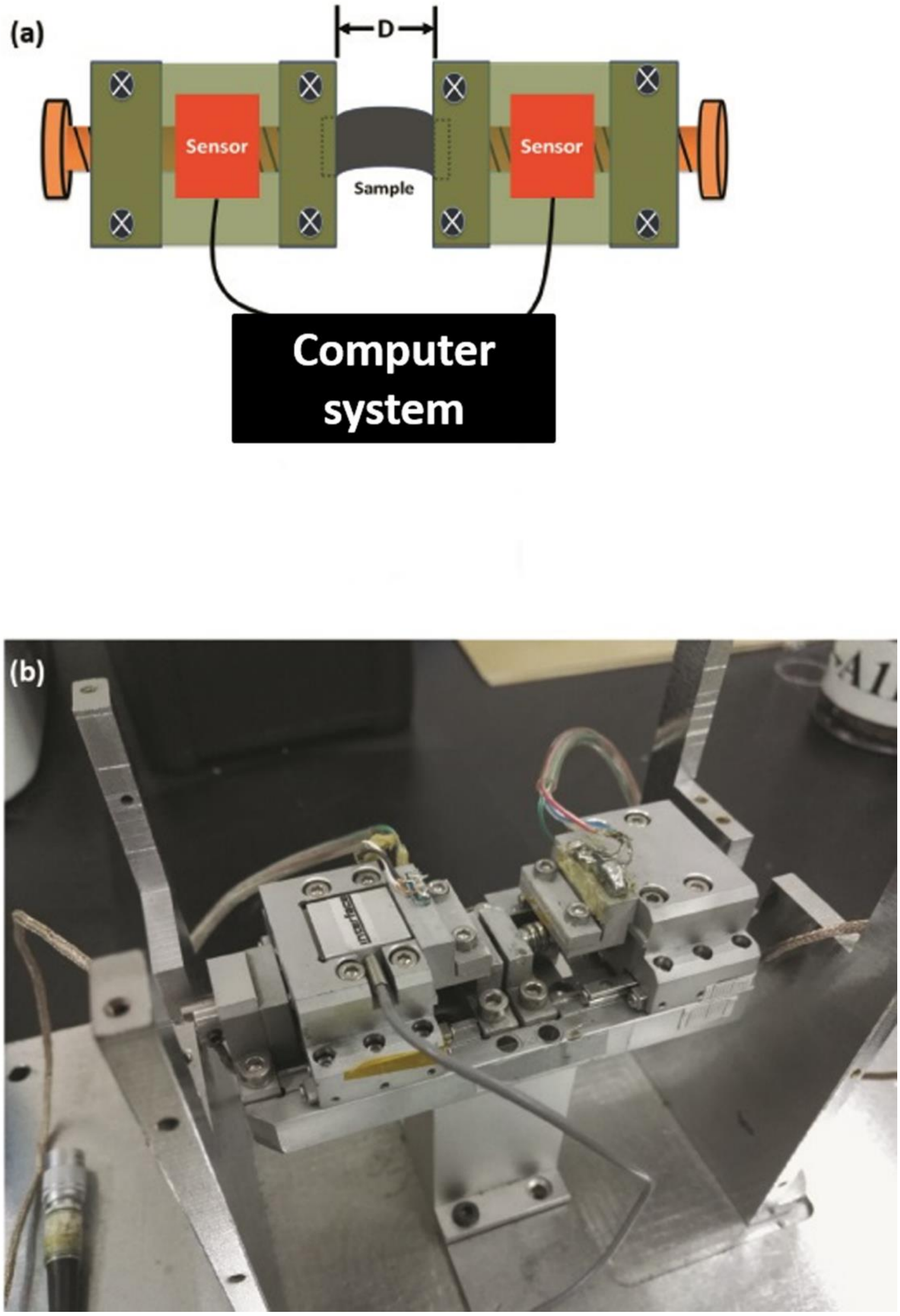

Supplementary Figure 5 │Instrumentations for electromechanical measurements. (a) Schematic illustrations and (b) digital image of the home-made apparatus for applying and measuring bending strains.

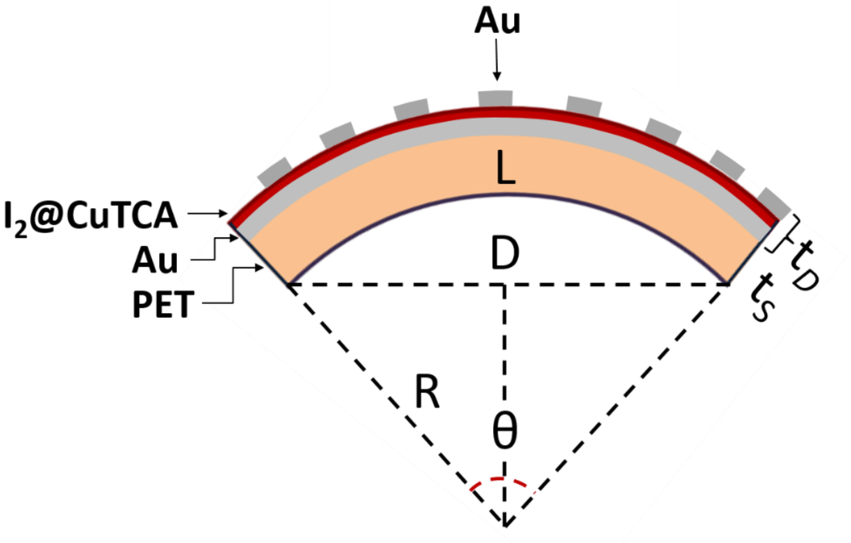

Supplementary Figure 6│Illustrative estimation of the surface strain. The Au/I_2@CuTCA/Au/PET device is not drawn to scale for the purpose of a better illustration.

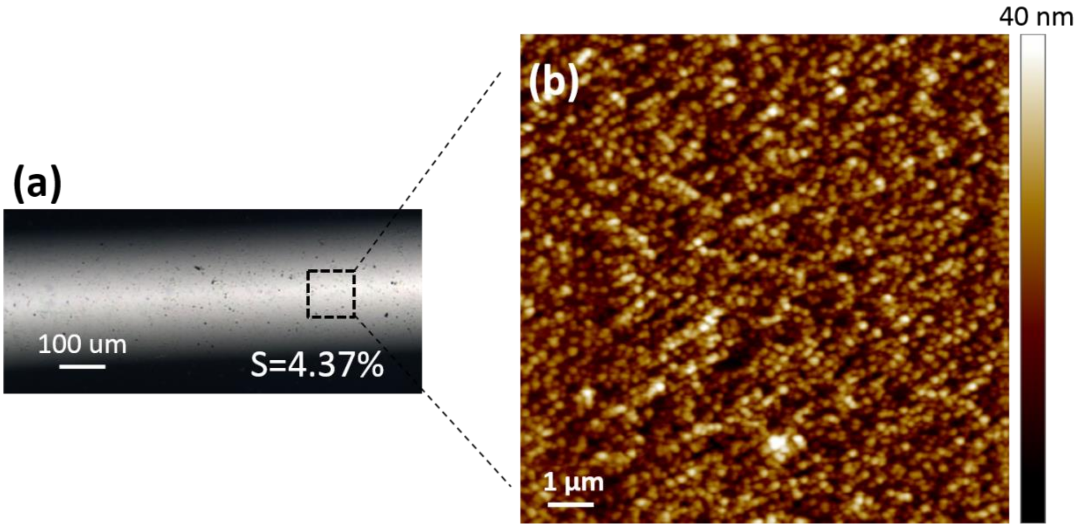

Supplementary Figure 7 | Morphology of the bended I_2@CuTCA nanofilm. The test was conducted on sample with the I_2 molecule/N atom ratio of 1.07:1 under bending strain of 4.37%, as observed through (a) an optical microscope and (b) an atomic force microscope, respectively. The bright region of the optical image corresponds to the bent part of the sample.

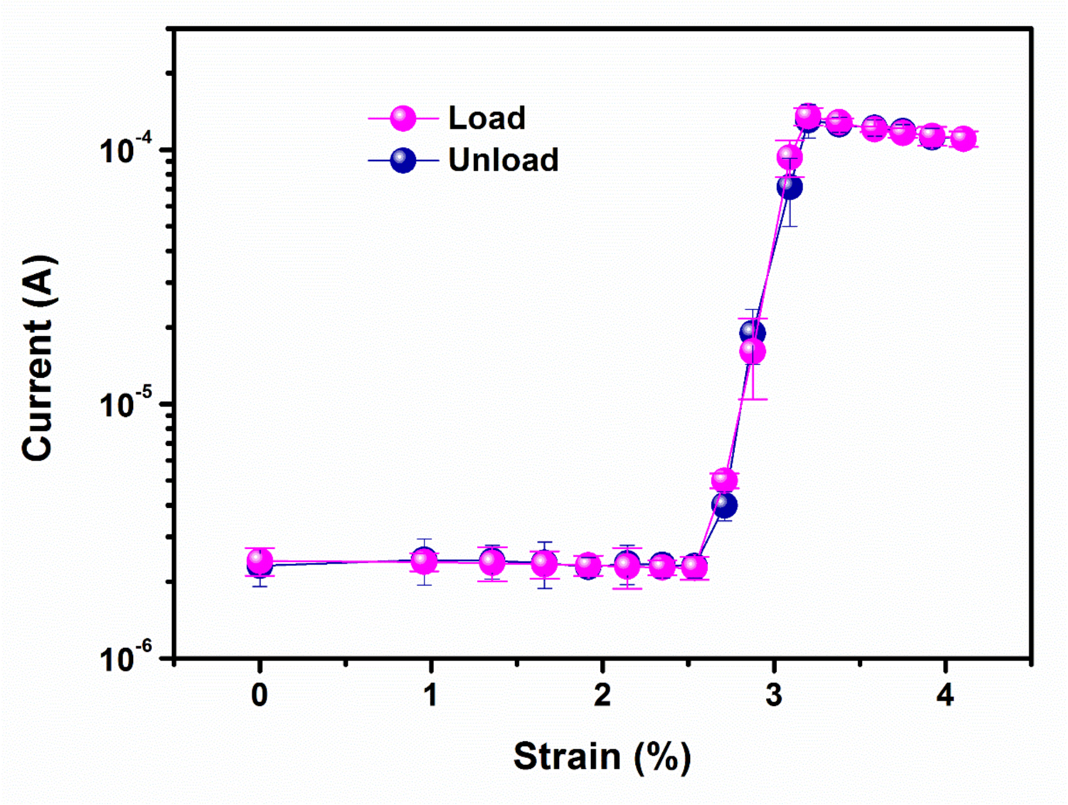

Supplementary Figure 8 | Electromechanical properties of the MOF film. The load-unload curve of the I_2@CuTCA nanofilm (with the I_2 molecule/N atom ratio of 1.07:1) device is tested in the strain range of 0% to 4.1%.

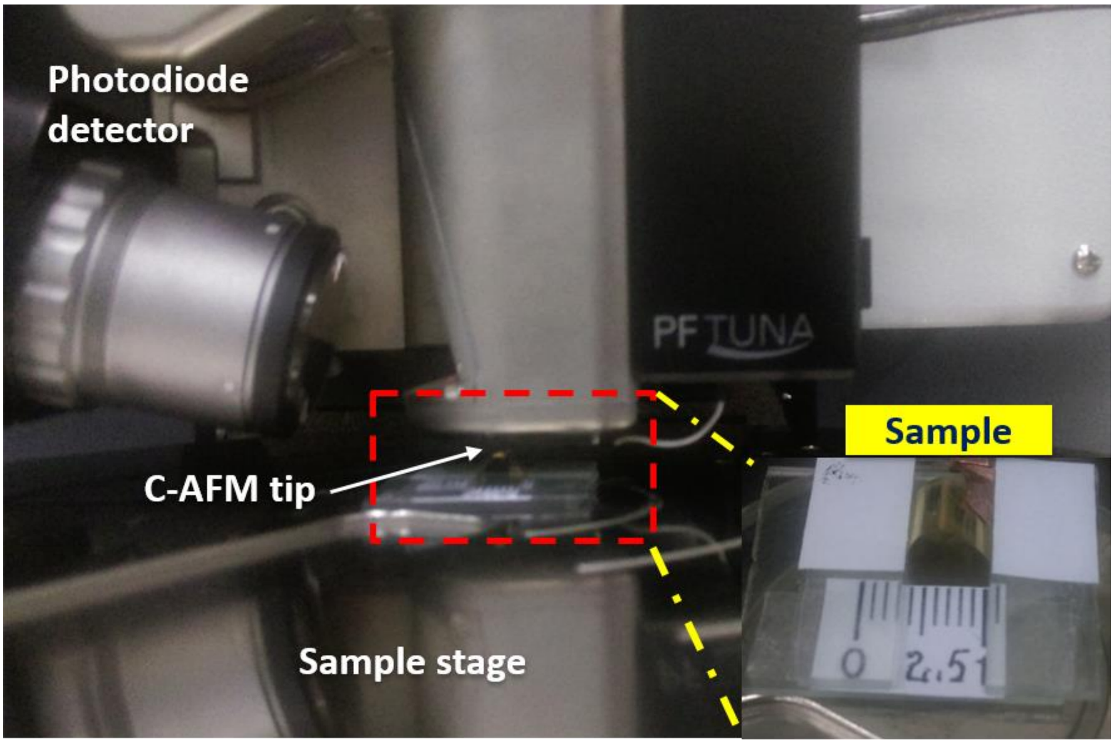

Supplementary Figure 9 │The experimental setup for C-AFM measurement. Inset shows the I_2@CuTCA nanofilm sandwiched between a Pt/Ir-coated conductive tip (grounded) and the Au/PET substrate.

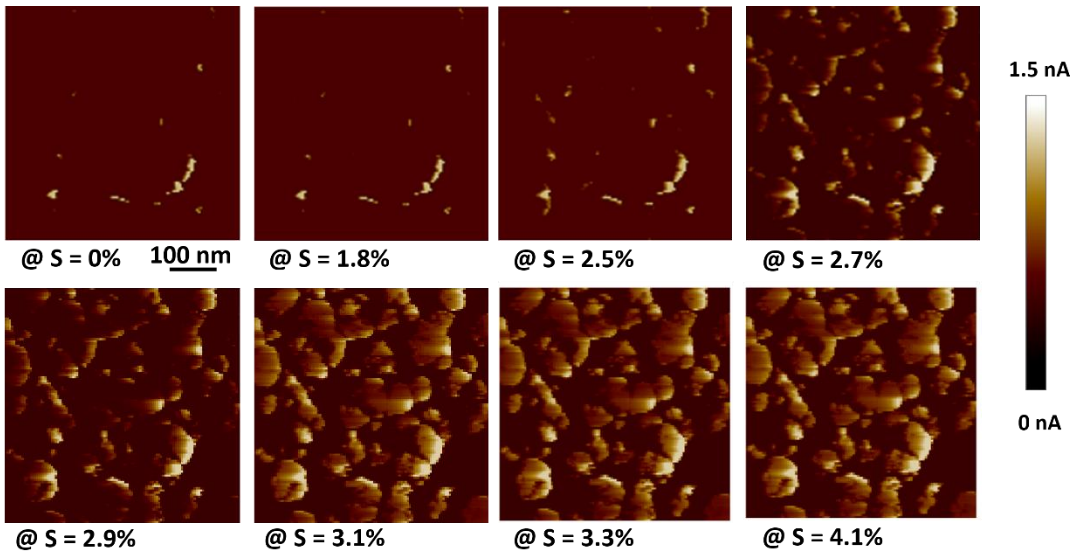

Supplementary Figure 10 │Current maps of the bended I_2@CuTCA nanofilm. The tests were conducted on samples with the I_2 molecule/N atom ratio of 1.07:1 under different bending strains of 0% to 4.1%, respectively.

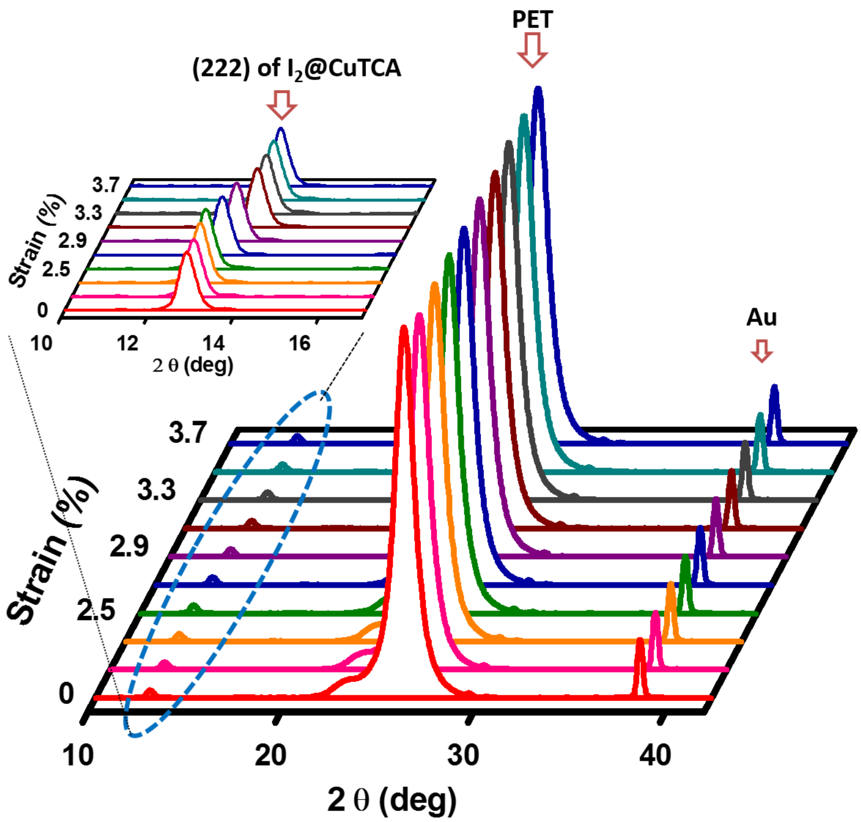

Supplementary Figure 11 │XRD spectra of the bended I_2@CuTCA sample. The tests were conducted under different bending strains of 0% to 4.1%.

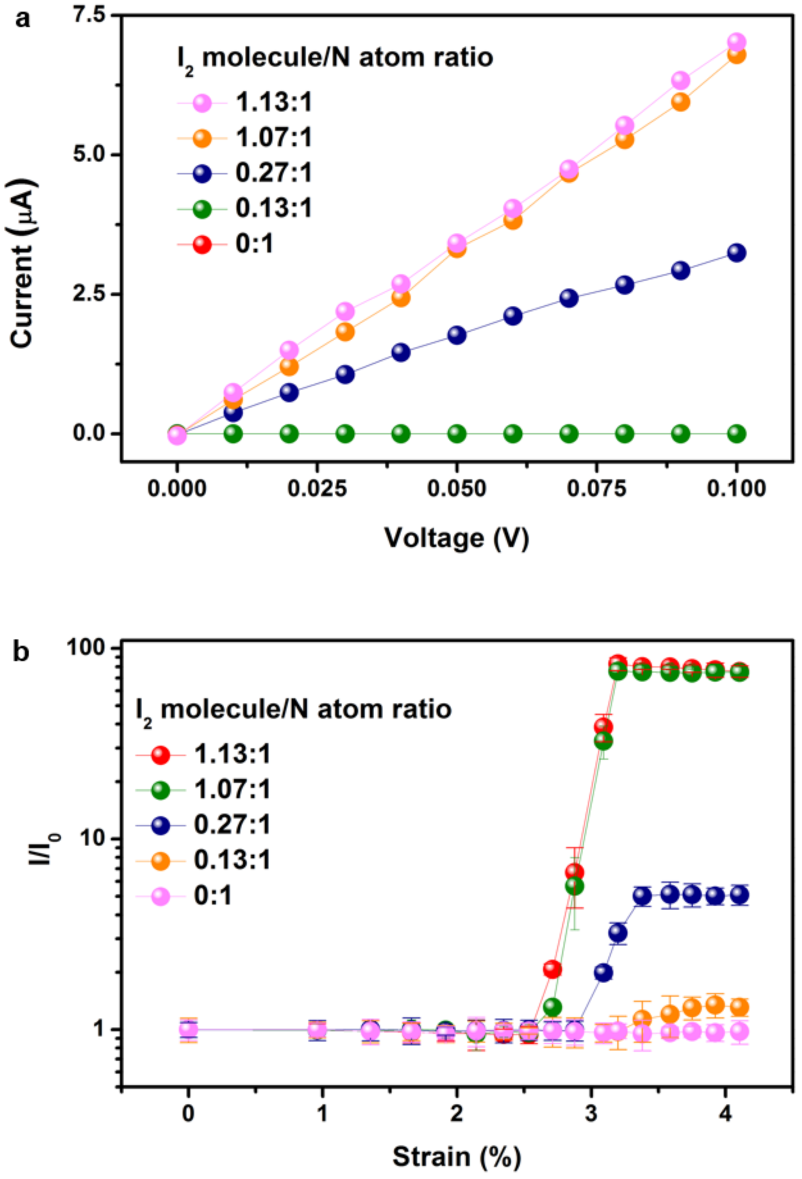

Supplementary Figure 12 │Electromechanical properties of doped samples. (a) Current-voltage characteristics of the unbended Au/I_2@CuTCA/ Au devices at different doping levels. (b) Electromechanical behavior of the Au/I_2@CuTCA/Au device with different doping levels.

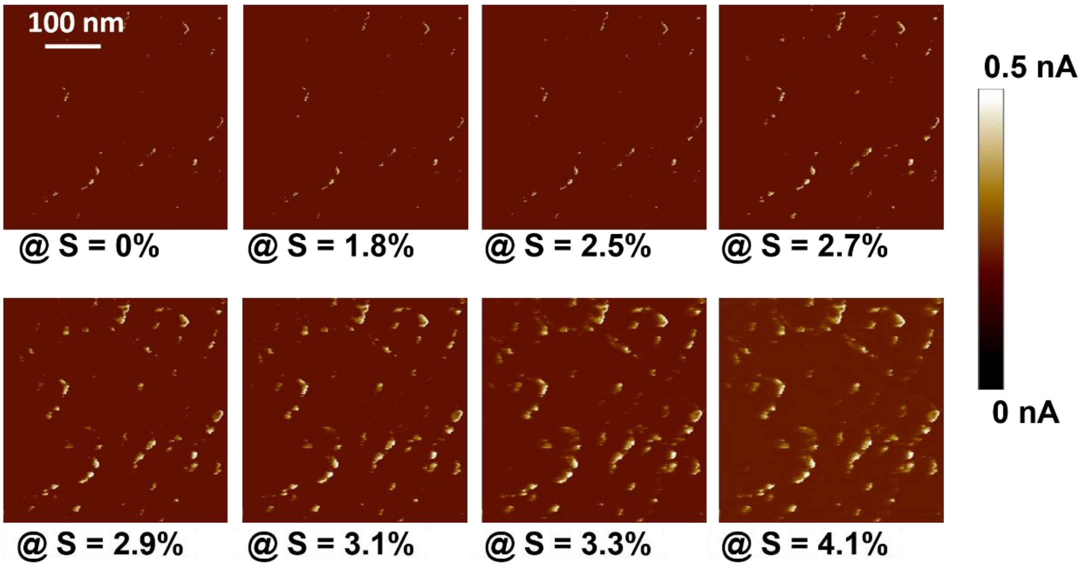

Supplementary Figure 13 │Current maps of the I_2@CuTCA nanofilm. The tests were conducted on samples with the I_2 molecule/N atom ratio of 0.27:1 under different bending strains of 0% to 4.1%, respectively.

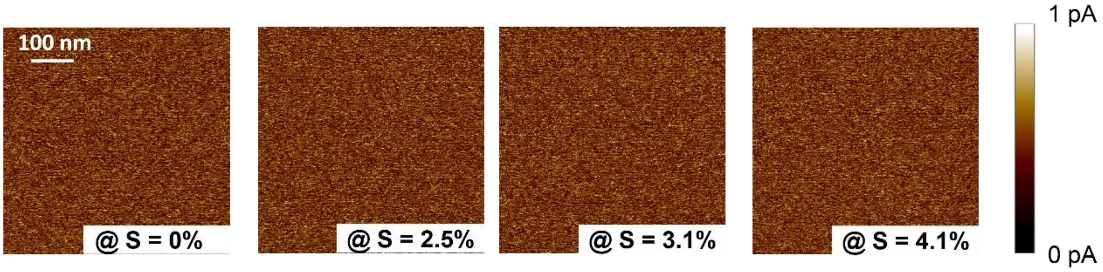

Supplementary Figure 14 │Current maps of the I_2@CuTCA nanofilm. The tests were conducted on samples with the I_2 molecule/N atom ratio of 0.13:1 under different bending strains of 0% to 4.1%, respectively.

Supplementary Table 1. Parameters for non-bonded and bonded interactions of CuTCA.

Atom	Type	Mass	Charge(e)	sigma (nm)	epsilon ($kJ \cdot mol^{-1}$)
Cu	Cu	63.54600	0.758	3.905e-01	7.32200e-01
N	N	14.0270	0.118	3.905e-01	4.93712e-01
C	CR1	12.0110	-0.130	3.905e-01	4.93712e-01
C(-N)	C	12.0110	-0.048	3.905e-01	4.93712e-01
C(-O)	C	12.0110	0.820	3.905e-01	4.93712e-01
O	OM	15.9994	-0.710	3.550e-01	1.04600e+00
H	HC	1.0080	0.158	0.000e+00	0.00000e+00

Supplementary Table 2. The bonded parameters for CuTCA

Bond			Angle			Dihedral		
	b_0 (nm)	K_b (kJ·mol^{-1}nm^{-4})		θ_0 (deg)	K_θ (kJ·mol^{-1})		θ_0 (deg)	K_θ (kJ·mol^{-1}rad^{-2})
CR1-HC	0.109	12300000	HC-CR1-CR1	120.0	505	CR1-C-CR1-HC	0.0	167.4
CR1-CR1	0.139	10800000	CR1-CR1-CR1	120.0	505	C-CR1-CR1-C	0.0	209.3
C-CR1	0.139	10800000	CR1-C-C	120.0	505	OM-C-C-CR1	180.0	5.9
C-C	0.139	10800000	C-C-OM	119.10	770	CR1-C-N-C	180.0	33.5
C-OM	0.125	13400000	OM-C-OM	121.78	770	C-N-C	117.0	635
C-N	0.133	10600000	OM-Cu-OM	89.34	380			
Cu-Cu	0.262	2.3900e+06	Cu-Cu-OM	83.81	380			
Cu-O	0.195	3.9700e+06	C-OM-Cu	125.40	575			
			CR1-C-N	120.76	505			
			C-N-C	117.0	635			

Supplementary Notes

Supplementary Note 1: Raman spectrum of CuTCA and I_2@CuTCA nanofilm

The Raman absorption peaks of CuTCA and I_2@CuTCA (**Figure 1d**) at the wave-numbers of 1614cm^{-1}, 1596 cm^{-1} and 1012 cm^{-1} are associated with the vibration of the C=C bonds of the benzene ring, while the peaks at 1176 cm^{-1}, 1143 cm^{-1} and 855 cm^{-1} are ascribed to the out-of-plane bending vibration of the C-H bonds. The absorption peaks at the wavenumber of 1427 cm^{-1} and 1395 cm^{-1} are arising from the asymmetric and symmetric stretching vibration of the carboxylic (-COO) groups of the BTC linkers, respectively. The Cu^{2+} species exhibit Raman absorption peaks at 411 cm^{-1} and 83 cm^{-1}, respectively. In particular, the absorption peaks at 1273 cm^{-1} and 1317 cm^{-1}, which are attributed to the stretching vibration of C-N bonds, shifts to 1251 cm^{-1} and 1295 cm^{-1} after introducing the guest I_2 molecules. A new peak of I_2 also appears at the wavenumber of 106 cm^{-1}. These results strongly illustrate that charge transfer interaction occurs between the infiltrated I_2 molecules and the CuTCA frameworks.

Supplementary Note 2: X-ray photoelectron spectroscopic characterization

The occurrence of charge transfer interaction between the infiltrated iodine molecules and the TCA linkers of the CuTCA framework was readily studied by X-ray photoelectron spectroscopic (XPS) measurements. As shown in **Supplementary Figure 2a**, the as- prepared undoped CuTCA sample demonstrates N 1s signal at the binding energy of 398.2 eV, which can be ascribed to the amine nitrogen atom of the TCA linker. After soaking the sample in iodine/ethanol solution with the I_2 concentration of 100 μM, obvious new nitrogen species with the binding energies of ~ 399.2 eV and 400.2 eV appear (**Supplementary Figure 2b**), which can be attributed to the neutral N atoms at the higher oxidative state (e.g. imine) and the positively charged N atoms upon the occurrence of charge-transfer interaction between the I_2 molecules and TCA linkers, respectively. The I 3d spectra of the I_2@CuTCA sample also show clear I $3d_{5/2}$ and I $3d_{3/2}$ signals at the binding energies of 619.2 eV and 360.6 eV, respectively, which are arising from the iodine species encapsulated inside the MOF framework (**Supplementary Figure 2c** and **2d**). Through calculating the XPS spectral area ratio of the iodine and nitrogen species in the I_2@CuTCA nanofilm, a 1.07:1 ratio of the I_2 molecule/N atom can be estimated in the samples obtained by soaking the MOF sample in iodine/ethanol solution with the I_2 concentration of 100 μM. The I_2/N ratios of the samples, which are prepared by soaking the pristine MOF nanofilms in iodine solutions with different I_2 concentrations, are estimated with the same approach.

Supplementary Note 3: Theoretical Simulations.

Molecular dynamics (MD) simulation was conducted on I_2@CuTCA to seek fundamental understanding of the interaction between the infiltrated iodine molecules with the MOF framework with the GROMACS 4.07 simulation package[1] and GROMOS96 force fields[2]. The crystal structure of CuTCA was downloaded from the Cambridge Structural Database (CSD) hosted by the Cambridge Crystallographic Data Centre (CCDC).[3] The molecule models employed in this study were generated from the small-molecule topology generator PRODRG.[4] During simulation, (2×2×2) unit cells of CuTCA were employed according to that revealed by X-ray diffractive analysis, while 64 molecules of iodine (according to an experimental 1:1 ratio of the I_2 molecule/N atom) were inserted near the N atoms in the nano-pores of the framework. The cut-off distance for short-range non-bonded interaction was chosen to be 12 Å and long-range electrostatic forces were computed using the proper electrostatic methods. After being set up, the entire system was first subjected to a steepest descent energy-minimization with a cut-off of 10 Å for van der Waals and Coulomb forces, then subjected to further thermalization by the constant-temperature MD runs at 300 K. The details of the force field parameters were listed in **Supplementary Table 1** and **2**. Bond lengths of molecules were constrained using the LINCS algorithm.

In MD simulations, two neighboring atoms interact with each other through van der Waals interactions, which is treated using a 12-6 Lennard-Jones (LJ) potential summed over all pairs of atoms i and j. The LJ potential may also be written in the following form:

$$V_{LJ}(r_{ij}) = 4\varepsilon_{ij}((\frac{\sigma_{ij}}{r_{ij}})^{12} - (\frac{\sigma_{ij}}{r_{ij}})^{6}) \tag{1}$$

where r_{ij} is the distance between the interacting pairs of atoms, σ_{ij} and ε_{ij} are the LJ parameters between atoms. The GROMACS LJ potential parameters $C_i^{(6)}$ and $C_i^{(12)}$ can be defined using the combination rules:

$$C_i^{(6)} = 4\varepsilon_i \sigma_i^{6} \tag{2}$$

$$C_i^{(12)} = 4\varepsilon_i \sigma_i^{12} \tag{3}$$

The combinations for different atom-types can be computed according to the combination rule:

$$C_{ij}^{(6)} = (C_i^{(6)} C_j^{(6)})^{\frac{1}{2}} \tag{4}$$

$$C_{ij}^{(12)} = (C_i^{(12)} C_j^{(12)})^{\frac{1}{2}} \tag{5}$$

The density-of-states information of the iodine doped CuTCA was calculated by DFT calculations, performed with Perdew-Burke-Ernzerhof (PBE) functional within the generalized gradient approximation using Gaussian 09 code.[5-7] The lanl2dz basis functions is applied to the system for Cu and I atoms.[8-11] The b3lyp/6-31+g(d,p) is applied to the system for C, O, H and N atoms. The structures of the simulated I_2@CuTCA system under 0% and 4% strains were chosen from the specific snapshots of MD simulation results as shown in **Figure 2a**.

Supplementary Note 4: Estimation of Device Strains

The bended surface of the device is assumed as an ideal part of a perfect circle at all bending radius,[12,13] while the slight deviation from the perfection may lead to some acceptable error for bending radius estimation. The strain S induced on the surface of the bended device can be estimated from following the equations:

$$S = \frac{(t_D + t_s)(1 + 2\eta + \chi\eta^2)}{2R(1+\eta)(1+\chi\eta)} \tag{1}$$

$$\chi = {Y_D}/{Y_S} \tag{2}$$

$$\eta = {t_D}/{t_S} \tag{3}$$

where t_D and Y_D are the thickness and Young's modulus of the device, t_s and Y_S are the thickness and Young's modulus of the PET substrate, and R is the curvature radius of the sample upon being bended. Since the thickness of the device is much smaller (t_D = 80+100+200 = 380 nm) than that of the PET substrate (t_S = 175 μm), equation (1) can be simplified as

$$S \approx {t_S}/{2R} \tag{4}$$

and the neutral plane of the entire sample is located in the PET substrate. As such, the MOF devices are experiencing tensile (stretching) strains when being bended upwards. Moreover, the strain across the entire device (e.g. at the top and bottom surface of the MOF layer) is almost uniform, which is also due to the much thinner thickness of the device in comparison to that of the substrate. Generally, the radius (R) of the curvature can be estimated from the following equations:

$$L = \theta \times R \tag{5}$$

$$\sin(\frac{\theta}{2}) = \frac{D/2}{R} \quad (6)$$

where L is the original length of flat device, θ is the angle of the curvature and D is the direct distance between the two sides of the sample upon being bended. Distance D can be directly measured through the home-made bending apparatus equipped with a pair of displacement sensors as shown in **Supplementary Figure 5**. With Taylor expansion the strain of the curved sample can be expressed as:

$$S = \frac{t_S}{L} \times \sqrt{\frac{6(L-D)}{L}} \quad (7).$$

Supplementary References

1. Van der Spoel, D. *et al.* GROMACS: Fast, flexible, and free. *J. Comput. Chem.* **26**, 1701-1718 (2005).

2. Oostenbrink, C., Villa, A., Mark, A. E. & Van Gunsteren, W. F. A biomolecular force field based on the free enthalpy of hydration and solvation: The GROMOS force-field parameter sets 53A5 and 53A6. *J. Comput. Chem.* **25**, 1656-1676 (2004).

3. Wu, P. Y. *et al.* Luminescent Metal-Organic Frameworks for Selectively Sensing Nitric Oxide in an Aqueous Solution and in Living Cells. *Adv. Funct. Mater.* **22**, 1698-1703 (2012).

4. Schuttelkopf, A. W. & van Aalten, D. M. F. PRODRG: a tool for high-throughput crystallography of protein-ligand complexes. *Acta Crystallogr. D-Biol. Crystallogr.* **60**, 1355-1363 (2004).

5. Perdew, J. P., Burke, K. & Ernzerhof, M. Generalized gradient approximation made simple. *Phys. Rev.w Lett.* **77**, 3865-3868 (1996).

6. Perdew, J. P., Burke, K. & Ernzerhof, M. Generalized gradient approximation made simple. *Phys. Rev.w Lett.* **78**, 1396-1396 (1997).

7. Frisch, M. J., et al. Gaussian 09, Revision B.01. 2009: Wallingford CT.

8. Dunning, T. H. D. Jr. and Hay, P. J. in *Modern Theoretical Chemistry, Ed. Schaefer, H. F.* III, Vol. 3: p. 1-28, Plenum, New York, 1976.

9. Hay, P. J. & Wadt, W. R. Abinitio effective core potentials for molecular calculations-potentials for the transition-metal atoms Sc to Hg. *J. Chem. Phys.* **82**, 270-283 (1985).

10. Wadt, W. R. & Hay, P. J. Abinito effective core potentials for molecular calculations-potentials for main group elements Na to Bi. *J. Chem. Phys.* **82**, 284-298 (1985).

11. Hay, P. J. & Wadt, W. R. Abinitio effective core potentials for molecular calculations-potentials for K to Au including the outermost core orbitals. *J. Chem. Phys.* **82**, 299-310 (1985).

12. Willian, S. W. and Alberto, S. Flexible Electronics: Materials and Applications, Springer-Verlag, New York 2009.

13. Han, S. T., Zhou, Y. & Roy, V. A. L. Towards the Development of Flexible Non-Volatile Memories. *Adv. Mater.* **25**, 5425-5449 (2013).
