# Supplementary material for: Mechano-regulated metal–organic framework nanofilm for ultrasensitive and anti-jamming strain sensing
Source: Nat Commun. 2018 Sep 19;9:3813. doi: 10.1038/s41467-018-06079-3 (PMC6145903; doi:10.1038/s41467-018-06079-3)
Supplement: Supplementary file 3 — Description of Additional Supplementary Files [file 41467_2018_6079_MOESM3_ESM.pdf]

Description of Additional Supplementary Files

Supplementary Movie 1

Description: Lattice geometry evolution of the I_2@ CuTCA crystallite under different strains. The frameworks of I_2@CuTCA gradually shrink with increasing applied strain. And the guest iodine molecules exhibit minor vibration around the nitrogen atoms of the TCA ligands, instead of travelling erratically inside the free MOF pore-space in a Brownian mode.

Supplementary Movie 2

Description: Real time monitoring of human walking with the kneecap based on I_2@CuTCA device. During 1 h walking of ~ 6238 steps (with the step width of 0.6 m/step) for a 65 kg male adult, a total walking distance of 3.74 km and energy consumption of ~ 198 kcal/828 kJ is achieved, equivalent to 0.03 calories per walking step.
